# Combined fiscal policies to promote healthier diets: Effects on purchases and consumer welfare

**DOI:** 10.1371/journal.pone.0226731

**Published:** 2020-01-15

**Authors:** Juan Carlos Caro, Pourya Valizadeh, Alejandrina Correa, Andres Silva, Shu Wen Ng

**Affiliations:** 1 Department of Health Policy and Management, Gillings School of Global Public Health, University of North Carolina, Chapel Hill, North Carolina, United States of America; 2 Carolina Population Center, University of North Carolina, Chapel Hill, North Carolina, United States of America; 3 Department of Economics, Universidad de Chile, Santiago, Chile; 4 Department of Economics, Government and Communication, Universidad Central de Chile, Santiago, Chile; 5 Department of Nutrition, Gillings School of Global Public Health, University of North Carolina, Chapel Hill, North Carolina, United States of America; Free University of Bozen-Bolzano, ITALY

## Abstract

Taxes on unhealthy foods and sweetened beverages, as well as subsidies to healthy foods, have become increasingly popular strategies to curb obesity and related non-communicable diseases. The existing evidence on the welfare effects of such fiscal policies is mixed and almost uniquely focused on tax schemes. Using the 2016-2017 Chilean Household Budget Survey, we estimate a censored Exact Affine Stone Index (EASI) incomplete demand system and simulate changes in purchases, tax incidence, and consumer welfare of three different policy scenarios: (1) a 5 percentage point additional tax on sweetened beverages (currently taxed at 18%) and a new 18% tax on sweets and snacks, (2) a healthy subsidy by zero-rating fruits and vegetables from the current 19% value-added tax, and (3) a combined (tax plus subsidy) policy. Under full pass-through of these policies, the combined scheme captures the incentives to switch purchases from both single-policy alternatives, resulting in a net welfare gain and subsidy transfer for the average Chilean household. In terms of welfare, low-income households strictly benefit from a combined policy, while high-income households experience a small consumer welfare loss, resulting in re-distributional effects.

## Introduction

The global prevalence of obesity has increased dramatically since 1980 [1]. More than one in two adults and nearly one in six children are overweight or obese in the OECD area [2], with increased risk of several non-communicable diseases (NCD) [3, 4]. In response to the obesity and NCD crises, governments have become increasingly interested in implementing fiscal policies to curb unhealthy food consumption. In recent years, over 40 locations including the United Kingdom, Saudi Arabia, Mexico, Finland, France, Chile, Peru, Ireland, South Africa, and cities across the U.S. have implemented or modified taxes on sweetened beverages or added sugar [5]. During the same period, other countries or states have discussed and/or implemented taxes on saturated fat or unhealthy foods, including Mexico [6], Denmark [7], the state of Kerala in India [8] and Hungary [9]. In addition, governments have also explored subsidy and/or tax earmarking options, such as the European Union subsidy scheme established in 2008 that provides free fruits and vegetables to children in schools [10]. Similarly, India and Egypt (among other countries) subsidize several healthy food groups, including pulses and other staple foods.

To date, most of the debate around food-related fiscal policies has focused on estimating their potential to change purchasing patterns towards improved nutritional health, based on simulated models or empirical evaluations. Simulations often estimate price elasticities of demand for different food groups and then project changes in purchases in response to different fiscal policy scenarios. Some studies also investigate potential health effects (e.g., changes in body mass index (BMI), prevalence of cardiovascular diseases (CVDs), prevalence of obesity and/or health care cost savings at the population level [11–14]. Fewer articles have extended the analysis to estimate the consumer and producer welfare effects of such food policies [15–18]. However, despite the growing interest in combined fiscal policies (i.e. implementing taxes on unhealthy foods and subsidies on healthy foods simultaneously), the heterogeneous effects on purchases and consumer welfare of such combined schemes remain unclear [19–21]. Few studies have measured the health effects of combined fiscal policies, and there is also limited evidence regarding the distribution of changes in welfare [14, 22, 23]. Theoretically, the combination of food taxes and subsidies can have larger impacts on average household purchases while minimizing welfare losses, relative to a tax only scenario. However, due to variation in price sensitiveness and mean consumption across socioeconomic groups, the distributional welfare effects of combined fiscal policies remain as an empirical question. From a public policy perspective, estimating welfare effects across different sub-populations (by income, for example) is essential as they have implications for tax equity and political feasibility.

This study builds on previous evidence by measuring the combined effect of food-based, health-oriented fiscal reform on household purchases and consumer welfare across different income groups. A combined food policy scheme could boost the difference in relative prices between healthy and unhealthy foods, while reducing the consumer welfare loss. Although we estimate changes in purchases across all food groups, our focus is to measure the change in consumer welfare due to a combined policy, in comparison to two single policies: a tax on unhealthy foods and a subsidy on healthy foods. For this purpose, we estimate the compensating variation for each household due to changes in prices. Compensating variation (CV) is a (money-metric) measure of a change in the household’s welfare due to a change in market prices (e.g., as a result of a tax or subsidy). Put differently, CV measures how much money a household needs to receive (or give away) to be as well-off after an increase (decrease) in price as they were before the price change.

Chile is a compelling case study for several reasons. First, as a recently declared high-income country, Chile experienced a rapid rise in disposable income in the last decades, therefore policy analysis can be informative for many middle and high income countries. Second, Chile has a high prevalence of obesity (34.4% among population 15 years and older in 2016) and type 2 diabetes (12.3% in 2016) [24], having the highest prevalence of overweight and obesity among OECD countries. Third, Chile recently introduced a comprehensive suite of regulations around food labeling on the front-of-package, restrictions of food marketing to children and an adjustment to taxation of beverages based on sugar content [25], which suggests interest in considering additional fiscal policy measures. Finally, the prevalence of obesity and related chronic diseases have been found to be higher among lower socio-economic status individuals, proxied by educational attainment [24]. Therefore, fiscal policies that discourage consumption of unhealthier options an/or encourage consumption of healthier options might benefit lower-income households to a larger extent.

We use the Chilean Household Budget Survey (2016-2017) to analyze the impact of three relevant fiscal policies in order to present results in context. First, a 18% tax on sweets and salty snacks and a further 5 percentage point increase to the already existing 18% tax on sweetened beverages (to bring the overall price change for SBs in Chile to 10% compared to before the systematic nutrition policy efforts in Chile started in 2012). Second, we consider a healthy subsidy by zero-rating fruits and vegetables from the current 19% value-added tax (VAT). Finally, we simulate the combination of both of these strategies. To simulate each scenario, we require food demand estimates. As such, we implement an incomplete censored demand system to determine households’ own and cross-price elasticities of demand for several food groups. In particular, we estimate an Exact Affine Stone Index (EASI) implicit Marshallian demand system, introduced by Lewbel and Pendakur [26]. The EASI demand system approach has two clear advantages for welfare estimation: Engel curves are not limited by functional form restrictions and error terms can be interpreted as consumer heterogeneity, similar to random effects. Ignoring such heterogeneity can cause important deviations on consumer welfare calculation [26]. Moreover, while ignoring household unobserved heterogeneity does not significantly affect estimated parameters, it can have substantial impact on welfare estimates [15, 26].

We find that the combined policy increases the average household’s fruits and vegetables purchases by 5.29 kilograms per month (1.33 and 3.96 kilograms respectively), decreases the average household’s sweets and snacks purchases by 1.43 kilograms per month, and reduces the average household’s sweetened beverage purchases by 0.9 liters per month. The combined policy creates an average welfare gain of 1.58 US dollar (USD) per month per household (0.08% of monthly income). Taxes and subsidies produce meaningful changes in household food purchases in the expected direction, where the larger changes, in absolute terms, occur among the highest income households (defined as the fifth income quintile). In relative terms (compared to baseline consumption), high income households also reduced sweetened beverage purchases more and increased fruit and vegetable changes more than low (defined as first income quintile) income households; there were no significant differences in relative purchase changes for sweets and snacks. Assuming complete pass-through of both taxes and subsidies to prices, the combined policy is estimated to create welfare transfers from highest income households (3.32 USD per month or 0.06% of monthly income) to lowest income households (0.99 USD per month or 0.23% of monthly income). Perhaps not surprisingly, our simulations based on a less than complete pass-through assumption translates to smaller losses/gains. As governments develop a stronger interest in fiscal policies to promote healthier purchases, we highlight the importance of considering combined strategies to address nutrition-related chronic diseases, not only to maximize the potential effects on consumer behavior, but also to mitigate welfare inequalities.

This article is organized as follows. The next section provides background on studies to date on fiscal policies on food purchases and consumer welfare. The third section describes the data and methods, then we present the results. The final section discusses the findings and their policy implications.

### Fiscal policies, food purchases, and consumer welfare

Despite current widespread adoption, food- or nutrient-based fiscal policies remain controversial means to curb obesity and nutrition-related chronic disease trends. From an economic perspective, price incentives can be justified as a way to internalize the externalities due to obesity and related chronic diseases on the health care system [27, 28]. Individuals with obesity have larger risk of chronic diseases such as cardio-vascular diseases and diabetes that lead to higher health care costs, driving up average prices for medical services [29, 30]. Obesity and related chronic diseases also generate indirect costs through lower labor market productivity due to lower labor force participation and amount worked by individuals as well as their caregivers [31]. Fiscal policies targeting certain foods or nutrients have also been argued as instruments to facilitate individuals to address *internalities*, i.e. the long-term individual costs associated to current poor nutritional diet [32]. Food and beverage taxation, particularly tax schemes based on the amount of critical nutrients of concern (such as sugar, sodium or saturated fats) per unit of volume or weight, can create incentives for product reformulation, improving the average nutritional quality of the food supply [33]. Finally, additional fiscal revenues from taxes can be used to fund public health initiatives [34] and to compensate for undesired distributional effects from such policies [19].

A key element of the global trend on food and beverage taxation is the emphasis on a rigorous evidence-based approach [5, 35]. Such evidence often is translated on estimated changes on average household purchases, based on simulated models or empirical evaluations. Few articles have extended the analysis to estimate the economic welfare effects of food policies, with mixed results. Yaniv, Rosin and Tobol [36], have developed a theoretical model that simulates a joint policy: taxes on unhealthy food and subsidies for food inputs (under the assumption that healthy foods are often cooked, thus also requiring time inputs). Authors find that such a strategy could decrease welfare for healthier individuals with a higher opportunity cost of time. Lusk and Schroeter [37] derived a simple model for high versus low calorie food demand, with utility explicitly depending on weight and physical activity. Based on their assumptions and previous results from other studies, they have concluded that for taxes to be welfare-increasing, the willingness to pay for weight reduction is remarkably high, around $1,500 per pound lost. However, this result depends strongly on the stability of the relationship between price increases and weight loss, which may vary depending on the tax size. Miao, Beghin and Jensen [38] have estimated welfare implications in a structural framework, aiming to calculate both consumer and producer welfare changes between a sales tax versus taxing inputs (e.g., sugar). They have found that taxing sweetener inputs is more efficient (lower surplus loss) than a sales tax, although strictly welfare reducing for consumers, as expected. On a similar note, Harding and Lovenheim [39] have demonstrated that nutrient-specific taxes are likely to produce lower welfare losses, given the larger tax base, compared to product-specific taxes. With respect to alternative policies, Allais, Etilé and Lecocq [16] have determined that mandatory labelling has a substantial differential effect on both welfare and purchases, compared to fat taxes. To date, there is only one study that explores the potential benefits of combining subsidies and taxes, reporting significantly larger health effects (compared to single policy strategies) but it does not present estimated changes in economic welfare [22].

In terms of heterogeneous welfare effects by income sub-groups, Chouinard et al. [40] have reported a large welfare variation in low-income households when fat taxes are applied to dairy products; however, their analysis ignores substitutions and complementarities with other food groups. Nnoaham et al. [14] shows that combined tax and subsidy policies to promote healthy diets are regressive, based on data from the United Kingdom. However, this study does not recognize that households from different income levels can have different underlying preferences. Zhen et al. [15] have revealed that sugar-sweetened beverage taxes would affect low-income households disproportionately in the United States, in a context where low-income households are also the high-consumer group. This is consistent with high-consumers being less sensitive to prices but displaying larger absolute decreases compared to average consumers when facing a tax schedule [41, 42]. In France, results based on simulation models indicate that taxing products high in sugar and fat are consistent with U.S. evidence [16]. Evidence from Germany also suggest that fat taxes create a higher burden on low-income households, despite reporting larger price elasticity [18]. Finally, Muller et al. [43] have provided experimental evidence in France suggesting that low-income women faced higher price increases due to taxes than high-income women and received less benefits from the taxes, due to both an unhealthier pre-tax base consumption and because of their lower responsiveness to the price changes. In sum, welfare changes across socioeconomic sub-groups depend not only on price elasticity of demand but also on the initial level of consumption.

We contribute to the previous literature, by examining the heterogeneous welfare effects of combined (subsidy and tax) policies, using a demand system approach that allows for larger flexibility on the relationship between income and purchases, using Chile as a case study. We estimate the demand system at each quintile of the household income distribution separately, to recognize that different income groups might have different baseline consumption levels of foods and beverages. We focus on combined policies due to in their potential to minimize average consumer welfare change while creating a larger relative price differential, thus inducing a joint effect to reduce unhealthy and increase healthy purchases. As mentioned, we use an incomplete demand system approach, to recognize the importance of substitutions and complementarities across food groups. This is critical, since previous evidence suggests that households with a strong taste for sugar are expected to substitute sweetened beverages (SB) with other sweet energy-dense foods [15, 27, 44].

## Materials and methods

### Household income and budget survey

In our analysis, we use the 2016-2017 Household Income and Budget Survey (EPF, Spanish acronym), [45], which is an income and expenditure survey conducted by the National Institute of Statistics (INE, Spanish acronym) in Chile. The EPF is conducted every five years in major urban areas, representing 74% of the urban population, and has a probabilistic, stratified, two stage sampling design. The EPF collects data about quantities and expenditures on all items (i.e., food and nonfood) used to construct the Consumer Price Index weights as well as socioeconomic and demographic information of the households (used to define poverty lines, among other applications). It also provides information about the month and geographical block of the survey.

The EPF interviewed a total of 15,239 households during the 12-month period from July 2016 to June 2017. After excluding households with incomplete survey information, our final analytic sample includes 15,184 households. In addition to examining the pricing policy effects in the full sample, we explore the potential heterogeneity in the impacts of policies on different households along the household income distribution. That is, we examine how lower vs higher income households may respond differently to price changes. To do this, we create five sub-samples using the quintiles of the household income distribution, where lower quintiles refer to households with lower income and likewise for higher quintiles.

Table 1 shows the descriptive statistics for the full sample as well as for low- and high-income households defined as those with annual income within the first and fifth quintile of the household income distribution, respectively. In the full sample, the average household head has about 11 years of education, 56% of households are headed by a male, the average household size is about three people per household, and about 52% of households reside in Santiago (there are two representative zones identified in the survey: main capital, Santiago, and the rest of the country). Additionally, we see that low-income households have a mean income of 436 USD per month whereas high-income households report a 13.5 times larger mean income of 5880 USD per month (average exchange rate in the period: 660 CLP = 1 USD). The large income disparities across households highlights the importance of exploring the heterogeneous effects of pricing interventions on households’ purchases, tax burden/subsidy transfer, and welfare by their income level.

**Table 1:** Household descriptive statistics.

	Full Sample		Low Income (Quintile 1)		High Income (Quintile 5)	
	Mean	Ref. Household	Mean	Ref. Household	Mean	Ref. Household
Household head’s education (years)	11.45	12	9.01	10	15.48	17
	(6.15)		(5.80)		(4.31)	
Male household head (%)	0.56	1	0.420	0	0.70	1
	(0.50)		(0.49)		(0.46)	
Household size	3.18	3	2.45	2	3.51	3
	(1.62)		(1.39)		(1.60)	
Number of children under 18	0.78	1	0.617	1	0.84	1
	(1.02)		(0.97)		(1.03)	
Number of adults	2.39	2	1.83	2	2.66	2
	(1.12)		(0.84)		(1.19)	
Number of men in the household	1.49	1	1.05	1	1.71	2
	(1.06)		(0.93)		(1.04)	
Share of households in Santiago	0.52	1	0.51	1	0.53	1
	(0.50)		(0.50)		(0.50)	
Households Income (USD)	2037.89	1284.05	436.33	454.54	5880.94	4588.01
	(2580.19)		(181.99)		(4042.11)	

Notes: Weighted values using sampling weights. Income distribution was calculated using nationally representative weights. As a result, sub-samples do not contain the same number of observations. The reference household corresponds to a representative household with median values of all socio-demographic variables. Standard deviations are in parentheses.

In the EPF, food expenditures are classified into food to be prepared and/or consumed at home (FAH), and food away from (FAFH) home. For FAH, the EPF also includes expenditures, quantities, and the acquisition place of 251 food items (i.e., Classification of Individual Consumption by Purpose (CCIF, Spanish acronym) codes), excluding alcoholic beverages. Using the CCIF codes, we divide FAH purchases into 10 groups that combine food products with similar nutritional content: (1) fruits, (2) vegetables, (3) carbohydrates, (4) sweets, desserts and salty snacks (hereafter, referred to as sweets and snacks), (5) seafoods, (6) red meat and poultry, (7) animal and vegetable fats, (8) dairies, (9) sugary and artificially sweetened beverages (hereafter, referred to as beverages), and (10) coffee, tea and water. We also define a composite numéraire good which includes all other FAH items (e.g., condiments and sauces), FAFH, and nonfood items. We use this numéraire good in our incomplete demand system to represent all other goods and services and to obtain unbiased measures of welfare (see, [46] and [47]).

Table 2 reports descriptive statistics for the 10 FAH categories and the composite numéraire good. For both the full sample and sub-samples by household income, we report household expenditure, unit value, purchase quantity (in kilograms or liters), budget share, and share of zero purchase of each FAH category. In the full sample, red meat/poultry and carbohydrates are the two FAH categories with highest expenditures. We also see that seafoods and beverages have the highest and lowest unit prices, respectively. Further, we see that the 10 FAH categories account for about 22% of household’s total expenditure. Turning to the sub-samples by household income, we observe that high-income households spend more on every food group (except carbohydrates), purchase higher quantities, pay higher unit values, and spend a smaller share of their budget on FAH than low-income households, with almost all differences being statistically significant (exceptions include the quantity purchased of carbohydrates and the budget share of poultry and red meat, and animal and vegetable fats).

**Table 2:** Food group statistics by household income group.

	Expenditure	Unit Value	Quantity	Share	Zero
**Full sample (All Income)**					
Fruits	13.06	1.9	9.0	1.1	27.66
Vegetables	30.44	1.5	28.5	2.7	10.73
Carbohydrates	44.89	1.7	29.4	4.6	1.92
Sweets and Snacks	34.77	4.4	10.3	2.4	11.35
Seafoods	8.29	7.6	1.4	0.6	56.46
Red Meat and Poultry	47.81	5.9	8.8	4.0	19.35
Animal and Vegetable Fats	21.74	5.6	4.8	1.8	13.61
Dairies	33.18	1.9	36.7	2.6	8.64
Beverages	22.10	1.1	21.5	1.8	14.74
Water, Coffee and Tea	7.87	5.2	12.9	0.6	42.08
Numéraire	1346.27	-	-	77.8	0.00
**Low Income (Quintile 1)**					
Fruits	7.68	1.7	6.5	1.4	35.14
Vegetables	20.67	1.4	22.9	3.9	14.09
Carbohydrates	35.47	1.6	25.8	7.2	1.83
Sweets and Snacks	15.67	4.0	5.9	2.5	21.23
Seafoods	4.20	6.6	0.8	0.7	65.50
Red Meat and Poultry	29.01	5.3	6.1	5.1	24.49
Animal and Vegetable Fats	13.67	4.9	3.5	2.5	17.72
Dairies	19.16	1.7	28.3	3.3	13.30
Beverages	11.68	1.0	13.3	2.0	25.12
Water, Coffee and Tea	4.15	4.8	6.5	0.7	54.16
Numéraire	469.53	-	-	70.6	0.00
**High Income (Quintile 5)**					
Fruits	22.79	2.3	11.9	0.7	19.69
Vegetables	43.96	1.8	31.8	1.4	10.72
Carbohydrates	46.62	1.9	24.2	1.6	3.45
Sweets and Snacks	68.59	5.2	16.4	2.0	4.29
Seafoods	15.30	9.7	1.8	0.5	48.17
Red Meat and Poultry	66.85	6.9	10.2	2.1	18.38
Animal and Vegetable Fats	32.38	7.1	5.4	1.0	12.82
Dairies	53.65	2.3	42.7	1.7	7.34
Beverages	31.97	1.3	26.5	1.0	9.73
Water, Coffee and Tea	14.73	5.5	23.3	0.5	27.13
Numéraire	3320.55	-	-	87.6	0.00

Notes: Expenditures and unit values are expressed in US dollars (USD). Quantities are measured in kilograms or liters depending on each food group. Shares and zeroes represent the fraction of the total budget and households reporting no purchases for each food group, respectively. Wilcoxon-Mann-Whitney’s test was conducted to check whether there is a significant statistical difference between low income (quintile 1) and high income (quintile 5) households. We found that almost all variables have a significant statistical difference between the two income groups (quintiles 1 and 5). The only exceptions were the budget shares of red meat/poultry, animal/vegetable fats, and carbohydrates.

### The demand model

To simulate different policy scenarios, we need to estimate price elasticities of demand for the 10 FAH categories plus the numéraire composite good. In particular, we estimate an Exact Affine Stone Index (EASI) demand system, introduced by Lewbel and Pendakur [26]. The EASI demand system has significant advantages over other demand system models (e.g., Almost Ideal Demand System, AIDS). First, it allows Engel curves of any order, not being subject to the rank three limitation discussed by Gorman [48] (i.e., Gorman’s rank restriction: no matter how many Engel curves are in the model, they must be expressed as linear combinations of at most three functions of expenditure). Previous evidence shows that, in general, Engel curves are quite non-linear, and therefore imposing strong functional form restrictions can have significant effects on estimates of consumer demand [15]. Second, since the EASI demand system is based on cost functions, welfare calculations are quite straightforward. Finally, error terms in the model can be interpreted as unobserved consumer heterogeneity, similarly to random effects, which has been proven to significantly affect welfare calculations [26].

The EASI demand system is based on the standard consumer theory, assuming that households maximize their utility subject to a linear budget constraint and face a *J*-vector of prices *p* = [*p*_1_, …, *p*_*J*_], where *J* is the number of goods and the *J*^*th*^ good is a numéraire good. The household has a total expenditure *x* after choosing a bundle of goods that is described by the *J*-vector of budget shares *w* = [*w*_1_, …*w*_*J*_]. Therefore, let *x* = *C*(*p*, *u*) be the cost function that provides the minimum nominal total expenditure to attain a utility level *u*, given prices *p*. These implicit Marshallian demands are hybrid demand functions of Marshallian and Hicksian demands that provide a direct approximation of household utility level as a function of observables. This way, we have been able to estimate the trade-off between income, price changes, and utility. The model is specified as follows:

$$w_{jh}=\sum_{k=1}^{J}a_{jk}lnp_{kh}+\sum_{r=1}^{R}b_{jr}y_{h}^{r}+\sum_{l=1}^{L}g_{jl}z_{lh}+\varepsilon_{jh}$$ (1)

where *w*_*jh*_ is the budget share of good *j* for household *h*, *y*_*h*_ is the real household total expenditure (implicit utility), *z*_*lh*_ is the *l*^*th*^ exogenous demand shifter, *lnp*_*kh*_ is the log price index of *k*^*th*^ good, and *ε*_*jh*_ is a vector of unobserved preference or heterogeneity parameters. Finally, *a*_*jk*_, *b*_*jr*_, *g*_*jl*_ are structural demand parameters to be estimated. Note that *R* is the highest order of the polynomial *y*_*h*_ (third order, in our case) and *b*_*jr*_ defines the shape of the Engel curve. Following Lewbel and Pendakur [26], we construct *y*_*h*_ as:

$$y_{h}=lnx_{h}-\sum_{j=1}^{J}w_{jh}lnp_{jh}+0.5\sum_{j=1}^{J}\sum_{k=1}^{J}a_{jk}lnp_{jh}lnp_{kh}$$ (2)

where *x*_*h*_ is nominal total household expenditures and foods and other goods and services. Lastly, standard demand restrictions including adding up, homogeneity, and symmetry are imposed in the EASI model. Specifically, symmetry implies *a*_*jk*_ = *a*_*kj*_, while homogeneity requires $\sum_{k=1}^{J}a_{jk}=0$ for all *j* = 1, …, *J*. As a result, we are able to produce a complete elasticity matrix per household. This allows us to have a distribution of elasticity estimates across households.

### Endogeneity and censoring

There are two sources of endogeneity in the demand model in Eq (1). The first type of endogeneity is due to the presence of budget share *w*_*jh*_ on both left- and right-hand-side (as an element of *y*_*h*_) of Eq (1). This type of endogeneity has been found to have little effects empirically (see, e.g., [15, 26]) and can be easily corrected by using the average budget share of the *j*^*th*^ food category across all households ${\bar{w}}_{j}$ in construction of *y*_*h*_ in Eq (2) (see, [26]).

The second type of endogeneity which is more important is concerned with prices. Although the use of micro-level (e.g., household-level) data may rule out the price endogeneity due to supply-demand simultaneity [15], substituting unit values of the aggregated food and beverage categories—calculated as the ratio between the category-level expenditures and category-level physical quantities—for exogenous market prices could lead to biased estimates of price elasticities. This is because category-level unit values contain information on both market prices and households’ choices of food quality (e.g., across different cuts of meats) within aggregated food categories (e.g., meat, see [49]). Put differently, the unit value (*ν*) can be expressed as the product of the actual market price (*p*) and a quality index (*π*) which is often referred to as an “expensiveness” index (i.e., *ν* = *p* × *π*; see [49, 50]).

To the extent that quality choices are trivial (e.g., *π* = 1) or are non-trivial but uncorrelated with unobserved characteristics in the demand model, the estimated price effects will be unbiased. However, unobserved quality choices are likely present and correlated with prices. In other words, it is possible that households respond to price changes by adjusting the quality of their food purchases (e.g., switching to cheaper cuts of meat in response to a price increase), causing unit values to be endogenously determined with quantity demanded.

To address the unit-value bias, we construct household-specific category-level price indices to approximate quality-quantity substitutions within the aggregated food and beverage categories. Specifically, we construct the Superlative Fisher Ideal price index for each food/beverage category using the CCIF-level unit values as elements, as done elsewhere (e.g., [15], [51] and [52]). For FAH category *j* = 1, …, *J* − 1, household *h*, the Fisher Ideal price index is calculated as the geometric mean of the Laspeyers (*P*^*L*^) and Paasche (*P*^*P*^) price indices:

$$P_{jh}^{F}=\sqrt{P_{jh}^{L}\times P_{jh}^{P}}=\sqrt{\frac{\sum \nu_{kht}q_{k0}}{\sum \nu_{k0}q_{k0}}\times \frac{\sum \nu_{kht}q_{kht}}{\sum \nu_{k0}q_{kht}}}$$ (3)

where *ν*_*kht*_ and *q*_*kht*_ are the unit value and purchase quantity of the *k*^*th*^ CCIF food code purchased by household *h* in month *t*, respectively; and *ν*_*k*0_ and *q*_*k*0_ are the base unit value and quantity of the CCIF code *k* which are set at their national averages in the sample. Since *P*^*L*^ provides an upper bound of the true (but unobserved) cost of living (or the price of the aggregated food categories in our application) while *P*^*P*^ gives a lower bound estimate, in general, the relationship *P*^*P*^ ≤ *P*^*F*^ ≤ *P*^*L*^ holds. The price index for the compositie numéraire category *P*_*Jh*_ is calculated as the share-weighted average of the Fisher price for all food items not directly modeled in our demand system and the Chilean monthly consumer price index (CPI) less share-weighted average of the Fisher price indices for the 10 FAH groups modeled.

Some of the CCIF-level unit values are missing because not all households buy all food types during the survey month. Thus, we need to impute missing unit values for non-purchasing households. We impute these missing unit values using the predicted values from a regression of reported CCIF-level unit values on zone dummies, month dummies, CCIF code dummies, dummy variables for geographical blocks of households, interaction terms between zone and CCIF codes, between month and CCIF codes, between month and zone, and a vector of demographic variables including number of children between 12 and 18 years, number of adults, number of seniors in the household, number of male household members, household head’s gender, age, marital status, and education level, the logarithm of household size, and the logarithm of household income and its squared form, as done elsewhere (see, e.g., [15, 52, 53]).

Lastly, since we are using cross sectional data at the household level and want to have a relatively large number of food groups in order to only aggregate similar food items, we needed to handle censoring (i.e., corner solution or zero purchase). The original model specification by Lewbel and Pendakur [26] does not take censored data into account. Thus, we use the two-step approach developed by Hein and Wesseils [54] to account for censoring. The first step involves the estimation of a Probit model describing the sample selection. Estimates from the Probit model are then used to calculate the inverse mills ratio (IMR) defined as the ratio of the probability density function to the cumulative distribution function for the budget share distribution of each food group. In the second step, estimates of the parameters of interest are obtained using a modified version of the EASI demand model, defined as:

$$w_{jh}=\sum_{k=1}^{J}a_{jk}lnp_{kh}+\sum_{r=1}^{R}b_{jr}y_{h}^{r}+\sum_{l=1}^{L}g_{jl}z_{lh}+\delta_{j}IMR_{jh}+\xi_{jh},$$ (4)

where *ξ*_*jh*_ is a random error term with unknown distribution.

### Estimation and simulation procedure

We estimate the demand system in Eq (4) using an iterated linear method, given the sample size restrictions. Estimation details are provided in Pendakur [55]. Using the estimated parameters, we estimate mean Marshallian demand elasticities for all households (see S1 File for details) and use them to compute changes in the quantity demanded and tax burden/subsidy transfer for each of the 10 FAH groups under each of the three fiscal policy scenarios. Additionally, we compute the compensating variation (CV) for the household with median demographic characteristics (shown in Table 1) under each policy scenario, which can be interpreted as the income a household needs to receive in order to return to the original utility level after a price change (see S1 File for details). Using the equivalence scale, we predicted the welfare effects for the remaining households in the sample, in relation to the median household (this is the household with median demographic characteristics). The equivalence scale can be defined as a measure of the cost of living of a household of a given size and demographic composition, relative to the cost of living of a reference household, when both households attain the same level of utility or standard of living [56]. Finally, we obtained standard errors for all estimates via a bootstrap approach with 500 replications.

### Simulation scenarios

The EASI model allows us to predict changes in the quantity demanded, tax burden and consumer welfare under policy scenarios noted earlier. We chose policy scenarios with the aim of attaining more realistic values that might be informative for policy. Our first policy scenario does two things. First, it extends the current 18% tax rate on sweetened beverages (SBs) with more than 6.25 grams per 100 milliliter (implemented since October 2014) to unhealthy foods [25]. Unhealthy foods, similar to SBs, has been point out as a leading obesity determinant [44]. Second, it further increases the current 18% tax rate on SBs to 23% which translates to a tax of 10% compared to pre-October 2014 tax level of 13%. This makes the SB tax in Chile comparable to other taxes that have been implemented such as in Mexico and South Africa [57]. Our second policy of zero-rating the existing 19% VAT from fruits and vegetables represents a net reduction on the price for healthy foods (fruits and vegetables), which has been proposed before in other studies [14, 22]. This scenario is consistent with evidence that most households do not meet the dietary guidelines for consumption of fruits and vegetables [24]. Moreover, evidence notes that zero-rating goods is more effective to induce changes in retail prices compared to a tax exemption [58]. Our final policy combines the two earlier policies.

We assume that price changes due to these fiscal policies are fully transferred to consumers (100% pass-through). However, it is possible that the pass-through of taxes and/or subsidies might not be complete. For instance, using food data from Brazil, Politi and Mattos [59] found an asymmetric tax response. Moreover, a number of evaluation studies assessing the pass-through of SB taxes and other unhealthy food taxes from Mexico and Chile have found that the degree of pass-through depending on product type and size ranges from 40-110%. Thus, we chose 75% pass-through of taxes as a mid-point in sensitivity analyses. For the subsidy, empirical studies looking at the pass-through when Chile lowered the value-added tax for low-sugar beverages showed that these ranged from 56-85%. Additionally, previous studies have shown that prices respond more to increases (taxes) than to decreases in value-added taxes [60], so in the sensitivity analyses we chose two levels of subsidy pass-through at 75% and at 50%. Thus, as sensitivity analyses, we estimated compensating variations of the three policy scenarios under two sets of pass-through assumptions: 75% pass-through for both tax and subsidy, and 75% pass-through for tax and 50% pass-through for subsidy.

## Results

### Estimated price elasticities and changes in purchases

Estimated mean Marshallian own- and cross-price elasticities are presented in Table 3. In the supplemental appendix (S1 File) we present supporting information, including estimated mean Marshallian own- and cross-price elasticities for low- and high-income households. All estimated own-price elasticities have a negative sign and are statistically significant. Cross-price elasticities are also significant in most cases. Further, we see that own-price elasticity estimates are different between low- and high income households, with high-income households own-price elasticities being larger for all FAH categories except sweets and snacks. Given the larger share of FAH in the budget of low-income households (29.4% vs 12.4% among high income households, see Table 2), their less-elastic demands for FAH categories are perhaps expected.

**Table 3:** Mean Marshallian price elasticities, full sample.

	with respect to the price of										
	Fruits	Vegetables	Carbohydrates	Sweets and Snacks	Seafoods	Red Meat and Poultry	Animal and Vegetable Fats	Dairies	Beverages	Water, Coffee, and Tea	Numéraire
Fruits	**-0.788**	-0.139	-0.068	-0.009	-0.045	-0.017	0.045	0.028	0.045	-0.010	0.255
	(-30.71)	(-8.35)	(-3.77)	(-0.58)	(-3.31)	(-1.47)	(2.46)	(2.15)	(3.04)	(-0.91)	(278.62)
Vegetables	-0.076	**-0.745**	-0.145	0.041	-0.019	0.067	-0.002	-0.050	0.006	-0.013	0.476
	(-2.56)	(-16.25)	(-4.95)	(1.46)	(-0.80)	(3.11)	(-0.07)	(-2.30)	(0.22)	(-0.64)	(28.67)
Carbohydrates	-0.021	-0.093	**-0.626**	0.037	0.023	-0.077	-0.037	-0.045	0.015	-0.008	0.688
	(-0.45)	(-2.21)	(-10.46)	(1.02)	(0.84)	(-3.12)	(-0.89)	(-1.58)	(0.38)	(-0.30)	(273.20)
Sweets and Snacks	-0.009	0.034	0.028	**-0.752**	-0.074	-0.059	-0.001	-0.034	0.033	0.013	-0.101
	(-0.40)	(1.34)	(1.27)	(-22.22)	(-3.99)	(-3.60)	(-0.04)	(-1.82)	(1.41)	(0.77)	(-83.92)
Seafoods	-0.040	-0.034	0.036	-0.097	**-0.870**	-0.056	0.017	-0.016	0.019	0.001	0.117
	(-2.47)	(-2.17)	(2.85)	(-6.96)	(-38.30)	(-5.27)	(1.21)	(-1.47)	(1.28)	(0.12)	(116.73)
Red Meat and Poultry	-0.006	0.032	-0.082	-0.024	-0.020	**-0.914**	-0.067	0.060	0.060	-0.005	0.354
	(-0.16)	(0.86)	(-2.69)	(-0.73)	(-0.70)	(-21.74)	(-1.93)	(1.97)	(1.64)	(-0.16)	(152.53)
Animal and Vegetable Fats	0.035	-0.007	-0.090	0.007	0.018	-0.159	**-0.750**	-0.067	-0.070	0.004	0.477
	(1.51)	(-0.29)	(-4.30)	(0.35)	(1.16)	(-11.17)	(-19.73)	(-4.45)	(-3.27)	(0.25)	(450.18)
Dairies	0.016	-0.060	-0.090	-0.026	-0.010	0.112	-0.053	**-0.934**	-0.005	0.019	0.367
	(0.76)	(-2.97)	(-4.91)	(-1.30)	(-0.63)	(7.07)	(-2.75)	(-39.87)	(-0.24)	(1.22)	(249.13)
Beverages	0.035	0.004	0.011	0.048	0.019	-0.074	-0.071	-0.005	**-0.977**	0.011	0.354
	(1.86)	(0.18)	(0.59)	(2.49)	(1.20)	(-5.00)	(-3.32)	(-0.32)	(-31.10)	(0.77)	(295.15)
Water, Coffee, and Tea	-0.015	-0.037	-0.046	0.027	0.002	-0.025	0.003	0.037	0.016	**-0.973**	0.117
	(-1.75)	(-4.30)	(-5.63)	(3.42)	(0.28)	(-3.31)	(0.35)	(5.36)	(1.84)	(-118.57)	(198.20)
Numéraire	-0.001	-0.001	-0.004	-0.008	0.000	0.002	0.002	0.000	-0.000	-0.000	**-1.115**
	(-0.02)	(-0.03)	(-0.07)	(-0.22)	(0.00)	(0.06)	(0.05)	(0.01)	(-0.01)	(-0.01)	(-193.16)

Notes: *t*-values calculated based on bootstrapped standard errors (500 replications) are in parenthesis. Underlined estimates differ significantly from 0 at the 5% significance level.

**Fig 1:**
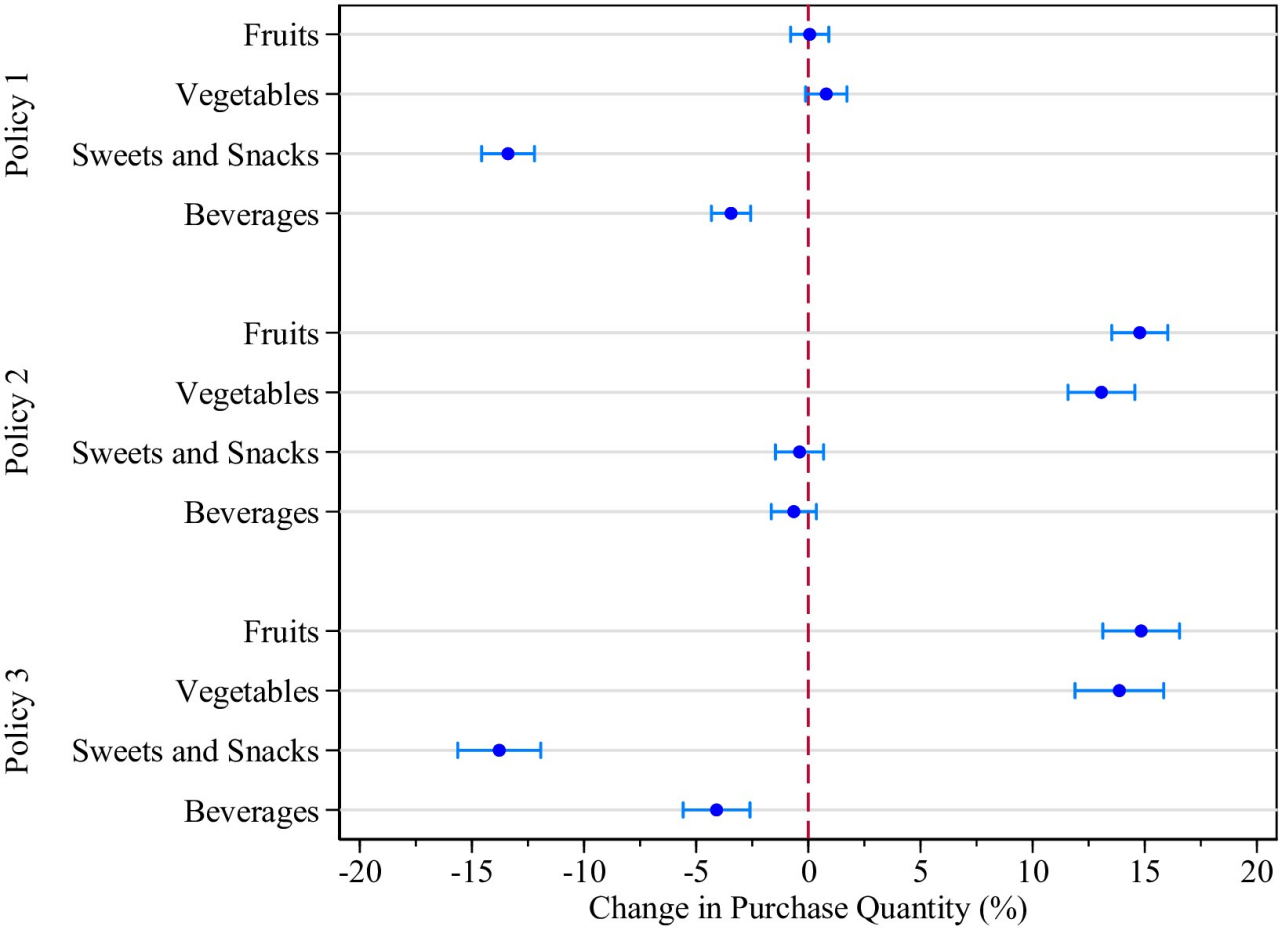
Average percentage change in household purchases (%). Notes: Results are shown only for food/beverage groups targeted by taxes or subsidies. All point estimates are accompanied by a 95% confidence interval calculated using bootstrapped standard errors.

Using the estimated price elasticities, we computed the purchase variation for each FAH group due to each fiscal policy. Estimated changes in purchases of targeted food/beverage categories in percentage terms for the full sample are shown in Fig 1. The absolute changes (in kilograms or liters per household per month) for all food groups, overall and by income quintile, are reported in Table 4. Our estimates reveal that an 18% tax on unhealthy foods and additional 5% point tax on SBs (Policy 1) would lead to an overall purchase decrease of 13.4% (1.39 kilograms) of sweets and snacks and 3.4% (0.7 liters) of beverages. There is also a slight substitution towards higher vegetable purchases of 0.8% (0.23 kilograms). A fruit and vegetable VAT zero-rating (policy 2) would lead to an overall increase of 14.8% (1.33 kilograms) of fruits and 13.07% (3.73 kilograms) of vegetables, with no significant effects on sweets and snacks or beverage purchase quantities. Finally, the combined policy incorporates both effects, almost in an additive fashion, as expected in a model linear in prices. Thus, increases in fruit purchases are estimated to be 14.84% (1.33 kilograms), vegetable purchase increases are estimated to be 13.87% (3.96 kilograms), while sweet and snack purchases are estimated to fall by 13.78% (1.43 kilograms) and SB purchases would decline by 4.09% (0.88 liters).

**Table 4:** Average change in household purchases in kilograms or liters per month.

	Full sample			Low Income (Quintile 1)			High Income (Quintile 5)		
	Policy 1	Policy 2	Policy 3	Policy 1	Policy 2	Policy 3	Policy 1	Policy 2	Policy 3
Fruits	0.00	1.33	1.33	-0.01	0.83	0.82	0.26	2.30	2.56
	(0.11)	(5.96)	(6.32)	(-0.22)	(2.74)	(2.81)	(2.00)	(4.58)	(5.55)
Vegetables	0.22	3.74	3.96	0.01	2.31	2.32	1.06	5.89	6.95
	(1.65)	(23.69)	(39.43)	(0.08)	(10.49)	(21.98)	(3.56)	(18.21)	(21.55)
Carbohydrates	0.21	0.53	0.75	0.29	0.90	1.19	-0.59	0.03	-0.56
	(1.79)	(9.59)	(98.54)	(1.88)	(14.80)	(169.82)	(-1.79)	(0.20)	(-21.36)
Sweets and Snacks	-1.39	-0.04	-1.43	-0.77	0.01	-0.76	-1.74	-0.47	-2.21
	(-21.11)	(-6.48)	(-29.87)	(-12.02)	(2.32)	(-13.89)	(-6.75)	(-26.96)	(-16.88)
Seafoods	-0.02	0.02	-0.01	-0.02	0.01	-0.01	0.00	-0.03	-0.02
	(-4.78)	(0.43)	(-0.18)	(-3.60)	(0.26)	(-0.10)	(0.12)	(-0.33)	(-0.27)
Red Meat and Poultry	-0.05	-0.04	-0.09	-0.04	-0.00	-0.04	0.01	-0.29	-0.29
	(-1.88)	(-1.42)	(-0.40)	(-1.27)	(-0.01)	(-0.13)	(0.09)	(-4.81)	(-0.59)
Animal and Vegetable Fats	-0.01	-0.02	-0.03	0.02	0.02	0.04	0.03	-0.18	-0.15
	(-0.38)	(-0.14)	(-0.19)	(0.85)	(0.09)	(0.25)	(0.43)	(-0.51)	(-0.31)
Dairies	-0.18	0.25	0.08	-0.29	0.20	-0.09	-0.18	-0.09	-0.27
	(-1.40)	(2.30)	(1.09)	(-1.79)	(1.69)	(-1.94)	(-0.64)	(-0.30)	(-0.77)
Beverages	-0.75	-0.13	-0.88	-0.42	0.02	-0.40	-1.82	-0.68	-2.50
	(-7.83)	(-2.65)	(-2.04)	(-4.50)	(0.61)	(-0.62)	(-5.55)	(-3.01)	(-2.32)
Water, Coffee, and Tea	0.07	0.11	0.18	-0.01	0.06	0.05	0.35	-0.06	0.29
	(1.86)	(1.35)	(0.39)	(-0.24)	(0.63)	(0.07)	(1.55)	(-0.24)	(0.31)

Notes: Each policy is defined in Table 5. *t*-values calculated based on bootstrapped standard errors (500 replications) are in parenthesis. Underlined estimates differ significantly from 0 at the 5% significance level.

**Fig 2:**
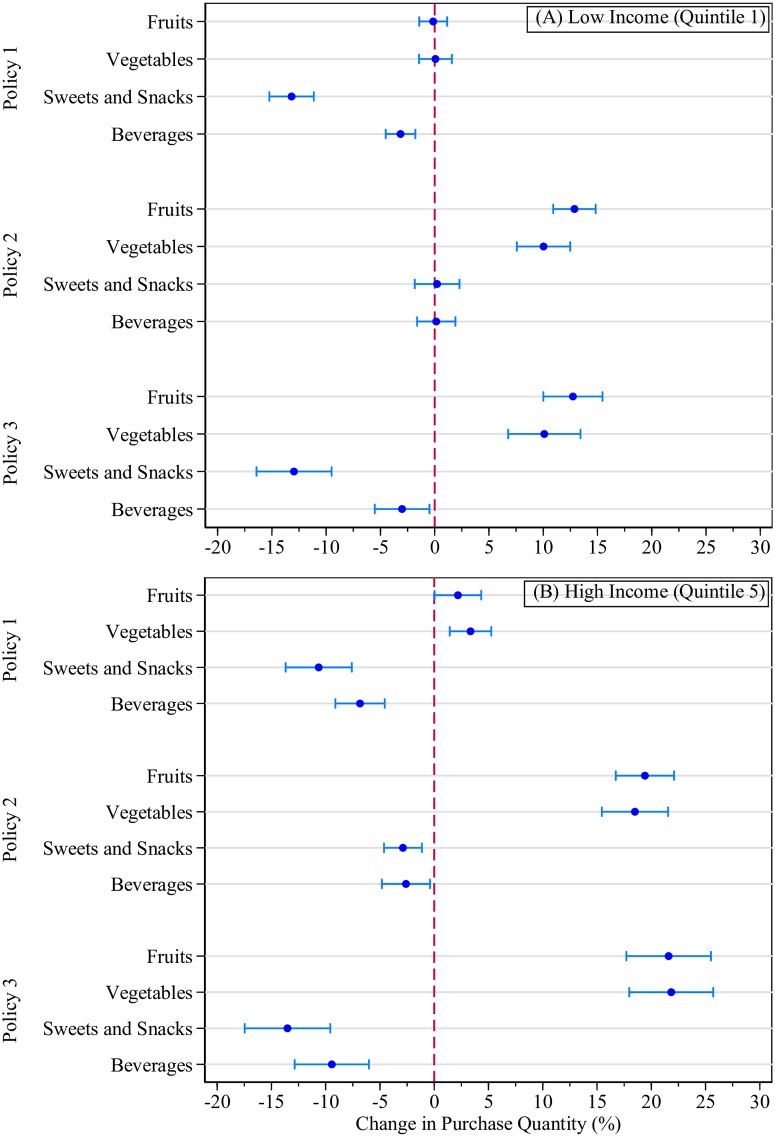
Average percentage change in household purchases (%), by household income. Notes: Results are shown only for food/beverage groups targeted by taxes or subsidies. All point estimates are accompanied by a 95% confidence interval calculated using bootstrapped standard errors.

Results by household income shown in Fig 2 suggest that high-income households show larger absolute changes (in kilograms/liters per household/month) in the purchase quantities of targeted foods and beverages than low-income households (Table 4). Given high-income households’ higher baseline purchase quantities and their more-elastic demands for FAH categories, these findings are expected.

### Estimated changes in welfare and tax burden or subsidy transfer

The welfare change (measured by compensating variation, CV) and tax burden estimates from different policies under the full (100%) price pass-through assumption, are presented in Table 5 in USD terms and as percentage of household income. The size of the tax burden and subsidy transfer from policies 1 and 2 are statistically different. As a result, the combined policy will result in a cost to the government of 2.83 USD per household (as an indirect transfer to households). In terms of welfare, households have a larger change in CV from the subsidies (9.69 USD), compared to the tax scheme (9.20 USD), and therefore the combined policy results in a net gain for the average household (0.48 USD). As expected, a tax policy would lead to an average household tax burden that is smaller than the welfare change in absolute terms. Implicitly, the difference is due to the dead weight loss of taxation.

**Table 5:** Estimated Compensating Variation (CV) and tax burden/subsidy transfer assuming a 100% pass-through of taxes/subsidies in prices, all households.

	Tax Burden/Subsidy Transfer		CV	
	USD	% of Income.	USD	% of Income
Policy 1: 18% Tax on Junk Foods + 5% additional tax on SB	8.08	0.40	9.20	0.45
	(103.30)	(75.31)	(113.00)	(97.68)
Policy 2: 19% Fruits/Vegetables VAT Reduction	-10.81	-0.53	-9.69	-0.48
	(-91.51)	(-70.79)	(-101.62)	(-93.57)
Policy 3: Tax and Subsidy Together	-2.83	-0.14	-0.48	-0.02
	(-16.89)	(-16.75)	(-5.20)	(-5.22)

Notes: *t*-values calculated based on bootstrapped standard errors (500 replications) are in parenthesis. Underlined estimates differ significantly from 0 at the 5% significance level. Positive values denote welfare losses/tax burdens whereas negative values denote welfare gains/subsidy transfers.

**Fig 3:**
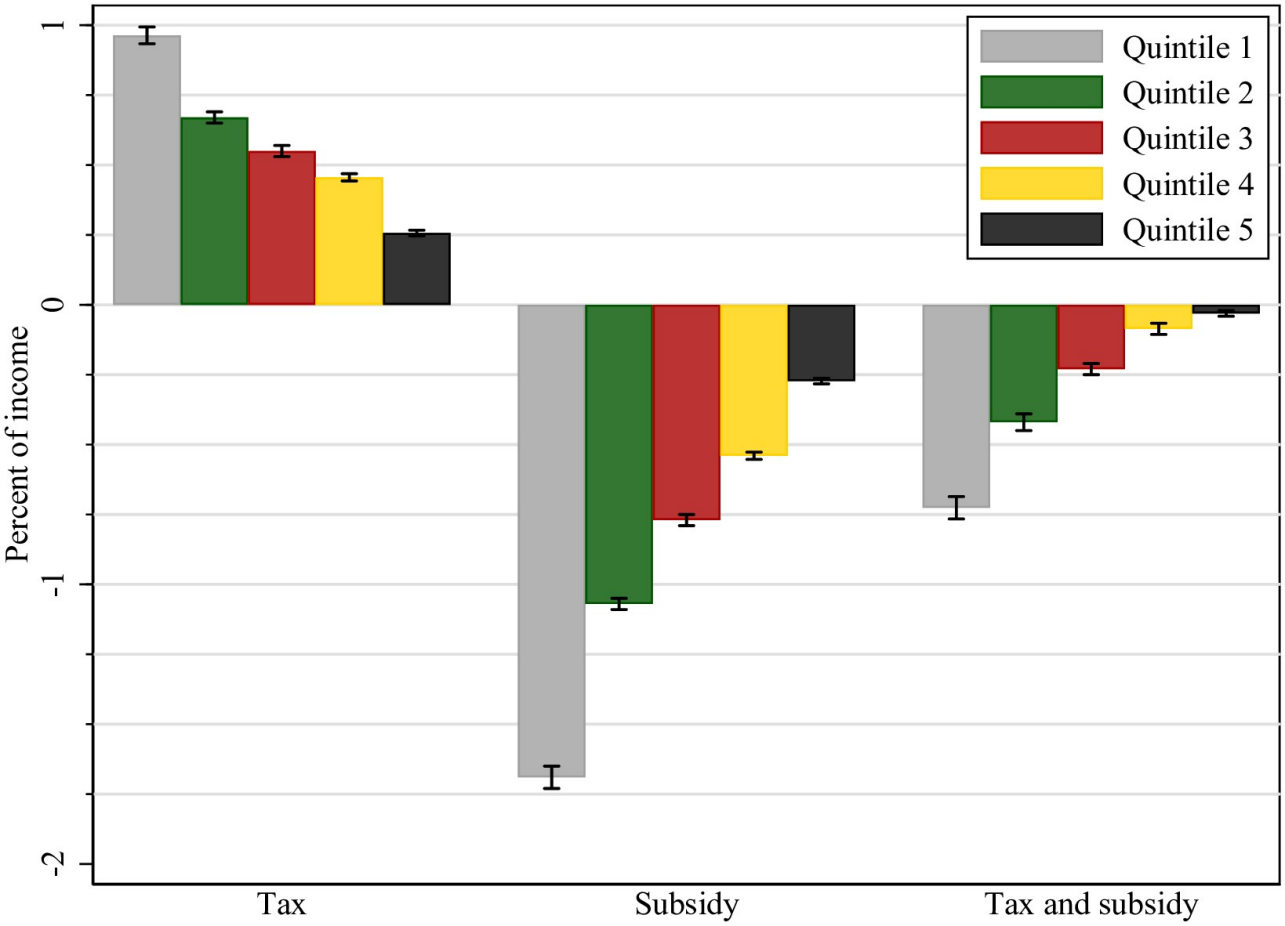
Household tax burden or subsidy transfer as (% of income). Notes: Positive values denote “tax burden” and negative values denote “subsidy transfers”. All values are expressed as percentage of household income.

**Fig 4:**
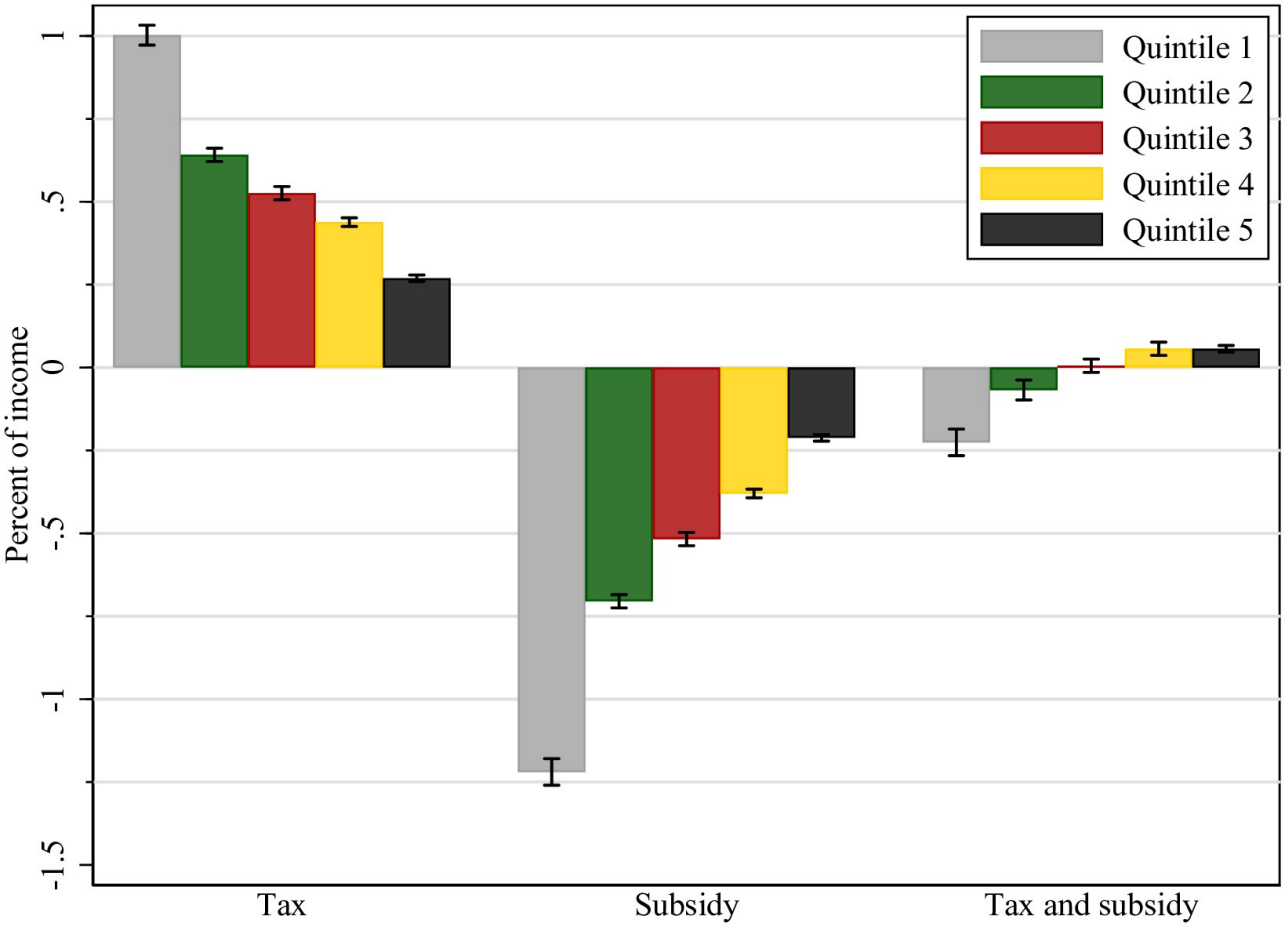
Household welfare change (compensated variation as % of income). Notes: Positive values denote “welfare losses” and negative values denote “welfare gains”. All values are expressed as percentage of household income.

Figs 3 and 4, and Table 6 present the CV and tax burden estimates by quintiles of household income distribution under complete pass-through of both tax and subsidy. There is significant heterogeneity in the tax burden of policy 1 between low- and high-income households. The lowest income (quintile 1) households report a much larger tax burden relative to their income, than highest income (quintile 5) households (0.96% versus 0.26%). In the case of a 19% VAT reduction on fruits and vegetables (policy 2), high-income households would capture a smaller subsidy transfer (relative to income), than low-income households (0.27% versus 1.69%).

**Table 6:** Estimated Compensating Variation (CV) and tax burden/subsidy transfer assuming a 100% pass-through of taxes/subsidies in prices, all households.

	Tax Burden/Subsidy Transfer		CV	
	USD	% of Income	USD	% of Income
Policy 1: 18% Tax on Junk Foods+ 5% additional tax on SB				
Quintile 1	4.21	0.96	4.36	1.00
	(42.58)	(42.24)	(56.15)	(53.25)
Quintile 2	6.33	0.67	6.03	0.64
	(45.19)	(45.35)	(58.03)	(58.33)
Quintile 3	8.07	0.55	7.64	0.52
	(41.31)	(40.98)	(57.81)	(57.50)
Quintile 4	10.47	0.46	10.04	0.44
	(42.50)	(42.63)	(62.15)	(63.48)
Quintile 5	15.10	0.26	15.81	0.27
	(45.17)	(40.20)	(58.67)	(53.76)
Policy 2: 19% Fruits/Vegetables VAT Reduction				
Quintile 1	-7.38	-1.69	-5.34	-1.22
	(-50.18)	(-50.70)	(-58.25)	(-56.74)
Quintile 2	-10.13	-1.07	-6.67	-0.71
	(-49.12)	(-48.96)	(-57.75)	(-57.42)
Quintile 3	-11.30	-0.78	-7.56	-0.52
	(-45.03)	(-45.42)	(-54.94)	(-55.09)
Quintile 4	-12.42	-0.54	-8.74	-0.38
	(-42.63)	(-42.83)	(-56.56)	(-56.34)
Quintile 5	-16.04	-0.27	-12.49	-0.21
	(-46.12)	(-42.17)	(-52.38)	(-49.77)
Policy 3: Tax and Subsidy Together				
Quintile 1	-3.16	-0.73	-0.99	-0.23
	(-15.43)	(-15.52)	(-10.12)	(-10.17)
Quintile 2	-3.97	-0.42	-0.64	-0.07
	(-13.63)	(-13.61)	(-5.23)	(-5.22)
Quintile 3	-3.39	-0.23	0.08	0.01
	(-8.83)	(-8.86)	(0.51)	(0.51)
Quintile 4	-1.97	-0.09	1.30	0.06
	(-3.66)	(-3.66)	(6.97)	(6.99)
Quintile 5	-1.82	-0.03	3.32	0.06
	(-3.20)	(-3.20)	(11.06)	(10.98)

Notes: *t*-values calculated based on bootstrapped standard errors (500 replications) are in parenthesis. Underlined estimates differ significantly from 0 at the 5% significance level. Positive values denote welfare losses/tax burdens whereas negative values denote welfare gains/subsidy transfers.

In the case of the combined policy simulation (policy 3), we found that both low and high-income households receive a net subsidy transfer. In terms of welfare changes, a tax policy will put a lower welfare cost to high compared to low income households, relative to their average monthly income (0.27% versus 1%). In the case of the VAT reduction policy, low income households receive a larger welfare benefit as a share of their income compared to high income households (1.22% versus 0.21%). The combined policy will create welfare transfers from high-income households (net welfare reduction of 0.06% income) to low-income households (net welfare gain of 0.23% income). Overall, we see that households within the top 40% of the income distribution experience a welfare loss from the combined policy, while households within lower 40% quintiles of the distribution experience a welfare gain from the combined policy.

Finally, we report the results of the sensitivity analysis in S1 File (Supplemental Appendix). First, we assumed equal 75% pass-through for both tax and subsidy, with the results shown by quintile of income. Secondly, we imposed a 75% pass-through on the tax, but 50% on the subsidy, based on the idea that tax cuts are less likely to be passed to final consumers [60]. We found that reducing the expected size of the pass-through have two clear welfare implications. If the degree of pass-through is similar but incomplete for both the tax and subsidy, then our conclusions remain unchanged; only the size of the effects are smaller. However, if the subsidy does not translate into lower prices in the same way as the tax (i.e. subsidy pass-through is smaller than tax pass-through), then the combined policy no longer creates welfare transfers between income groups. This is because all households regardless of income level will experience welfare loss. Although not presented here, expected reduction in purchases will also be reduced proportionally (linear) to the size of the expected pass-though on prices.

## Discussion

In this study we present the estimates of changes in purchases, tax burden (or subsidy transfer) and welfare of three different policy scenarios: taxes on unhealthy foods and beverages, a removal of an existing VAT for fruits and vegetables, and a combination of these two fiscal policies. In order to do this, we used the EASI demand system developed by Lewbel and Pendakur [26]. We estimated a set of elasticities to simulate quantity, tax burden (or subsidy transfer) and welfare losses (or gains) under these three fiscal policy scenarios. We estimate the effects using the full sample, and also at every quintile of the income distribution, recognizing that households at different quintiles might express different preferences, as noted in the previous literature.

As a per-household average, the combined policy would lead to an increase of 5.3 kilograms of fruits and vegetables per month. If we assume that one average portion of fruits and vegetables corresponds to 80 grams, and given the average household size, the combined policy would lead to an increase of 0.7 portion of fruits and vegetables a day per person. Low-income households in this sample are less price responsive (except for sweets and snack) than high income households and thus increase their fruit and vegetable consumption less than high-income households. This may be because the large income disparity means that low-income household spend considerably more of their budget on these 10 food categories (29.4%) compared to high income households (12.4%). Nonetheless, while we found that the changes are greater for higher income households in Chile, among the low income households (quintile 1), monthly fruit and vegetable consumption is still estimated to increase 3.1 kilograms, which translates to an increase of 0.4 portion of fruits and vegetables a day per person. As a comparison, Capacci and Mazzocchi [34] found that a public information campaign to promote fruits and vegetables consumption in the UK (5-a-day campaign) led to an increase of between 0.2 and 0.7 portions of fruits and vegetables a day. Furthermore, the combined policy reduces the consumption of unhealthy food by 1.4 kilograms and consumption of beverages by 0.9 liters, consistent with previous evidence [44].

A key issue of note in this study is our estimation of the welfare losses of gains under the three policy scenarios, and how this may vary by income quintile. We found that low-income households experience the largest relative welfare loss and gain (as share of income) for the tax and subsidy correspondingly (likely due again to the larger share of their budget on FAH compared to high income households). We also found that the combined policy results in welfare transfers from high-income households (loss of 0.06% income among fifth quintile) to low-income households (gain of 0.23% income among the first quintile). These results suggest that a combined policy can be one way for governments to not only improve dietary choices (and resultant health outcomes) of their population, but also helps redistribute resources to address equity concerns, without requiring direct transfers. It is notable that in our results, the welfare effects between the first and second quintiles are significantly different given that at the time of the survey, poverty (measured using a comprehensive set of indicators beyond income) in Chile reached nearly 20%.

To date, there are very limited studies that compare the welfare implications of various fiscal policy designs as applied to foods or nutrients to compare our findings to. Nnoaham et al. [14] is the closest study that presents a similar analysis, finding that a policy schedule in line with our combined policy scenario will be regressive, while our results suggests otherwise. However, this study uses a model that is linear in income and ignores differences in price elasticities across income groups. Such differences can account for the diverging conclusions.

Härkänen et al. [22] consider a sugar tax, a tax reduction for healthy foods and a combined policy in Finland on changes in energy and nutrient intake and extrapolate this to provide a range of estimates on health outcome changes. Akin to our findings, they found that there are significantly larger health effects with a combined policy compared to either single policy strategies, but their study did not estimate economic welfare changes. Härkänen et al. [22] also found that the health effects were the most pronounced for low-income individuals, primarily because low-income individual have the most severe health problems to start with. Consequently, the authors conclude that these policies may help reduce health inequalities in Finland. Extrapolating our findings to potential changes in the health, productivity and reduced health care expenditures that could arise from the three policies and how they might vary across income quintiles are beyond the scope of this paper. However, under all three scenarios, given increases in fruit and vegetable consumption and reductions in sugary beverages, sweets and snacks we should expect that the health and productivity effects and reduced health care expenditures should be strictly positive and sizable. Whether these impacts are disproportional between income groups remains as a empirical question for future research. However, given that the prevalence of obesity-related non-communicable diseases is larger in low socioeconomic groups, we can expect similar effects of health inequalities as presented in Härkänen et al. [22] and Ministery of Health [24].

Our work presents some limitations. First, we were only able to analyze demand for foods and beverages, we do not consider other substitution and income effects beyond this set of purchases, based on the assumption of staged budgeting, as noted in other studies [15, 44]. Second, due to data limitations, we were not able to distinguish artificially sweetened beverages from sugar-sweetened beverages and were only able to look at sweetened beverages collectively. We also worked under the assumption of a uniform pass-through across household income groups. In one of the few studies on tax pass-through, Colchero et al. [57] found that the soda tax in Mexico has a different pass-through on price across beverages and regions. Thus, it could be the case that some income groups do not respond to price variations, as determined by Bertail and Caillavet [61]. Third, we do not have actual measures of dietary intake, but rather only have measures of purchase behavior, so caution is needed for interpretations on what our finding might mean for obesity and other health related outcomes. Fourth, in terms of our approach to deal with unit-value bias, we acknowledge that to the extent that our Fisher Ideal price index construction uses CCIF-level unit values (in the absence of more disaggregated food classification such as brand-level data), price endogeneity may not be completely accounted for and some bias may remain.

Finally, in this framework, we have no means of providing information to compare the social benefits of the combined policy with other food-based welfare policies that represent a direct cash transfer and can simultaneously impact obesity prevalence, such as the Chilean School Meal Program [62]. Likewise, we have ignored the potential environmental dimension; unhealthy food and sweetened beverage production leads to larger greenhouse emissions than fruits and vegetables production. In France, Caillavet, Fadhuile and Nichèle [63] found a beneficial synergy between environmental and nutritional effects across income and age groups, with a small regressive impact. In this sense, future work can explore the effects at the nutrient-level as well as potential environmental impacts, as proxy to health effects. In any case, we argue that both tax incidence and welfare impact are relevant dimensions to consider as part of the debate around food and nutrition related fiscal policies. Without estimates of potential health, environmental and labor productivity benefits from shifting away from unhealthier foods towards healthier ones, our analytic approach can only provide an economic welfare estimation based on current purchase levels; we are unable to quantify the welfare gain attributable to healthier purchases in the long run.

As governments are increasingly exploring fiscal policies to promote healthier purchases, we highlight the importance of considering multiple strategies to address this goal, as well as consider the welfare changes and tax incidence overall and across income groups and how these policies may alleviate or worsen inequities. Our findings suggest that a combined policy taxing less healthy foods at 18%, increasing the tax on sweetened beverages to 23%, along with a subsidy/removal of the existing 19% VAT on fruits and vegetables in Chile is one potential way to improve dietary choices while also achieving redistributive goals.

## Supporting information

S1 File Supporting Appendix. (PDF)
