# Supplementary material for: Combined fiscal policies to promote healthier diets: Effects on purchases and consumer welfare
Source: PLoS One. 2020 Jan 15;15(1):e0226731. doi: 10.1371/journal.pone.0226731 (PMC6961856; doi:10.1371/journal.pone.0226731)
Supplement: S1 File — (PDF) [file pone.0226731.s001.pdf]

Suplementary Appendix

Price Elasticities

Following the procedure by Zhen et al. [15], we calculated the compensated (Hicksian) elasticity of demand for good j with respect to the price of good k and expenditure elasticities. Then, using the Slutsky equation, we computed uncompensated (Marshallian) price elasticities (dropped 7 out of 10,485 households/observations with extreme values).

Compensated (Hicksian) elasticity of demand for good j with respect to price of good k is,

$$\xi = \varpi^{-1}A + \Omega\varpi - I \tag{5}$$

where ξ is a JxJ matrix of compensated demand elasticities, ϖ is an identity matrix where the ones are replaced by the food group's budget shares, Ω is an JxJ matrix of ones and I is an identity matrix. Expenditure elasticities are calculated as:

$$\eta = \varpi^{-1}(I + BP')^{-1}B + 1_j) \tag{6}$$

where η is an J vector of expenditure elasticities and B is the derivative of equation 4 (model corrected by Shonkwiler and Yen [63]) with respect to the real expenditures y_l, whose jth elements equal $\sum_{r=1}^{3} rb_{ir}y^{r-1}$, P is the $JX1$ vector of log prices, and 1_j is a $Jx1$ vector of ones.

Uncompensated (Marshallian) price elasticity is recovered from the Slutsky equation,

$$E = \eta - \xi * S \tag{7}$$

Welfare Analysis

Based on the cost function $C(p_0, u, z, \varepsilon)$, we evaluated the cost to an individual of a price change based on Pendakur [55]. In order to obtain that result, we used the log cost of living index as a consumer surplus measure for the price change from p_0 to p_1 which, for the cost function, is given by:

$$\ln\left(\frac{C(p_1,u,z,\varepsilon)}{C(p_0,u,z,\varepsilon)}\right) = \sum_{j=1}^{J} w_0^j(\ln p_1^j - \ln p_0^j) + \frac{1}{2}\sum_{j=1}^{J}\sum_{k=1}^{J} a^{jk}(\ln p_1^j - \ln p_0^j)(\ln p_1^j - \ln p_0^j). \tag{8}$$

The first term of the equation 8 is the Stone index for the price change, which captures the first order effects driven by expenditure shares. The presence of the second term allows one to explicitly model substitution effects (or second order effects). The welfare calculated by equation 8 is related to the reference household. However, the interpretation of the error terms as unobserved preference heterogeneity allows the estimation of a distribution of welfare, instead of only the average welfare effect.

Finally, based on Lewbel and Pendakur [26], we adjusted our welfare estimations with an equivalence scale for households with different demographic characteristics. These equivalence scales allow inter-household comparisons of the welfare effect. The equivalence scale is given by:

$$\ln E(p,u,z,e) = \sum_{t=1}^{T} g_t^j z_t + \sum_{t=1}^{T}\sum_{j=1}^{J} lnp^j(g_t^j z_t + \varepsilon^j) + \frac{1}{2}\sum_{j=1}^{J}\sum_{k=1}^{J} a^{jk} lnp^j lnp^k, \tag{9}$$

where d are the coefficients of demographic variables, z is the vector of demographic variables, p is the vector of price index and A are price coefficients.

Supplementary Tables and Figures

Tables S1 and S2 shows the Marshallian price elasticities for low (quntile 1) and high (quintile 5) income households. Table S3 presents the compensated variation by income quintile assuming different pass through scenarios.

Table S1. Mean Marshallian own- and cross-price elasticities, low income (quintile 1) households

	with respect to the price of										
	Fruits	Vege-tables	Carbo-hydrates	Sweets and Snacks	Seafoods	Red Meat and Poultry	Animal and Vegetable Fats	Dairies	Bev-erages	Water, Coffee, and Tea	Numéraire
Fruits	**-0.737**	-0.069	-0.080	-0.022	-0.067	-0.133	0.019	0.048	0.059	-0.002	0.147
	(0.047)	(0.029)	(0.028)	(0.028)	(0.019)	(0.020)	(0.030)	(0.025)	(0.026)	(0.016)	(0.002)
Vegetables	-0.038	**-0.590**	-0.300	0.013	-0.015	0.091	-0.036	-0.059	-0.038	-0.022	0.300
	(0.050)	(0.082)	(0.049)	(0.055)	(0.035)	(0.038)	(0.060)	(0.046)	(0.050)	(0.025)	(0.029)
Carbohydrates	-0.023	-0.192	**-0.496**	0.067	0.035	-0.034	-0.025	-0.040	-0.010	-0.012	0.422
	(0.070)	(0.072)	(0.116)	(0.069)	(0.039)	(0.046)	(0.071)	(0.055)	(0.067)	(0.035)	(0.006)
Sweets and Snacks	-0.020	0.007	0.094	**-0.743**	-0.093	-0.089	0.040	-0.072	0.043	-0.005	-0.147
	(0.036)	(0.041)	(0.035)	(0.058)	(0.028)	(0.028)	(0.041)	(0.031)	(0.039)	(0.021)	(0.003)
Seafoods	-0.056	-0.025	0.060	-0.099	**-0.841**	-0.080	0.021	-0.015	-0.027	0.002	0.108
	(0.023)	(0.024)	(0.019)	(0.026)	(0.039)	(0.017)	(0.024)	(0.021)	(0.021)	(0.014)	(0.002)
Red Meat and Poultry	-0.049	0.050	-0.060	-0.038	-0.034	**-0.776**	-0.107	0.070	0.070	-0.015	0.064
	(0.054)	(0.061)	(0.052)	(0.061)	(0.040)	(0.075)	(0.059)	(0.055)	(0.058)	(0.039)	(0.005)
Animal and Vegetable Fats	0.017	-0.054	-0.076	0.049	0.022	-0.240	**-0.652**	-0.093	-0.055	-0.004	0.335
	(0.037)	(0.042)	(0.034)	(0.038)	(0.024)	(0.026)	(0.064)	(0.031)	(0.037)	(0.019)	(0.003)
Dairies	0.032	-0.076	-0.099	-0.059	-0.012	0.137	-0.079	**-0.907**	0.008	0.023	0.209
	(0.037)	(0.039)	(0.032)	(0.035)	(0.025)	(0.029)	(0.038)	(0.054)	(0.037)	(0.023)	(0.003)
Beverages	0.051	-0.060	-0.046	0.054	-0.027	0.018	-0.059	0.011	**-0.929**	0.007	0.168
	(0.030)	(0.033)	(0.031)	(0.035)	(0.023)	(0.024)	(0.035)	(0.029)	(0.050)	(0.018)	(0.002)
Water,coffee and tea	-0.004	-0.052	-0.058	-0.007	0.002	-0.055	-0.009	0.039	0.008	**-0.938**	0.105
	(0.013)	(0.012)	(0.011)	(0.013)	(0.009)	(0.011)	(0.013)	(0.012)	(0.013)	(0.012)	(0.001)
Numéraire	-0.001	0.000	-0.013	-0.011	0.002	-0.009	0.003	0.000	-0.001	0.001	**-1.099**
	(0.055)	(0.059)	(0.077)	(0.063)	(0.053)	(0.053)	(0.065)	(0.065)	(0.061)	(0.040)	(0.012)

Notes: Bootstrapped standard errors (500 replications) are in parentheses.

Table S2. Mean Marshallian own- and cross-price elasticities, high income (quintile 5) households

	with respect to the price of										
	Fruits	Vege-tables	Carbo-hydrates	Sweets and Snacks	Seafoods	Red Meat and Poultry	Animal and Vegetable Fats	Dairies	Bev-erages	Water, Coffee, and Tea	Numéraire
Fruits	**-0.922**	-0.294	-0.064	0.089	0.017	0.249	0.159	-0.030	0.131	-0.005	0.064
	(0.055)	(0.038)	(0.044)	(0.029)	(0.029)	(0.028)	(0.043)	(0.024)	(0.037)	(0.031)	(0.001)
Vegetables	-0.178	**-0.981**	0.027	0.177	0.062	0.167	0.060	0.037	0.036	0.011	0.127
	(0.063)	(0.096)	(0.075)	(0.042)	(0.048)	(0.040)	(0.062)	(0.038)	(0.054)	(0.048)	(0.038)
Carbohydrates	-0.036	0.030	**-0.709**	-0.136	-0.034	-0.116	-0.195	-0.009	0.010	-0.018	0.994
	(0.074)	(0.076)	(0.130)	(0.062)	(0.062)	(0.049)	(0.072)	(0.041)	(0.069)	(0.059)	(0.003)
Sweets and Snacks	0.042	0.138	-0.116	**-0.572**	0.015	0.023	0.023	-0.022	-0.078	0.030	-0.119
	(0.059)	(0.053)	(0.078)	(0.086)	(0.052)	(0.045)	(0.071)	(0.037)	(0.062)	(0.054)	(0.002)
Seafoods	0.013	0.080	-0.050	0.024	**-0.971**	-0.005	-0.002	0.024	-0.071	0.044	0.097
	(0.036)	(0.036)	(0.046)	(0.031)	(0.037)	(0.027)	(0.046)	(0.024)	(0.038)	(0.027)	(0.001)
Red Meat and Poultry	0.087	0.095	-0.068	0.020	-0.001	**-1.170**	-0.068	0.145	0.145	0.026	0.516
	(0.081)	(0.070)	(0.087)	(0.064)	(0.064)	(0.098)	(0.082)	(0.054)	(0.080)	(0.070)	(0.003)
Animal and Vegetable Fats	0.130	0.081	-0.264	0.042	-0.001	-0.161	**-0.814**	0.043	-0.048	0.025	0.521
	(0.053)	(0.046)	(0.054)	(0.043)	(0.046)	(0.035)	(0.076)	(0.030)	(0.050)	(0.043)	(0.002)
Dairies	-0.017	0.031	-0.013	-0.024	0.017	0.227	0.027	**-1.028**	0.003	0.022	0.175
	(0.044)	(0.042)	(0.045)	(0.033)	(0.036)	(0.033)	(0.043)	(0.044)	(0.039)	(0.042)	(0.002)
Beverages	0.112	0.051	0.013	-0.129	-0.071	-0.148	-0.049	0.009	**-1.019**	-0.004	0.891
	(0.045)	(0.039)	(0.049)	(0.036)	(0.036)	(0.033)	(0.048)	(0.026)	(0.059)	(0.041)	(0.002)
Water, Coffee and Tea	-0.006	0.022	-0.043	0.086	0.074	0.098	0.041	0.053	-0.008	**-1.106**	0.180
	(0.023)	(0.022)	(0.027)	(0.020)	(0.017)	(0.018)	(0.026)	(0.017)	(0.026)	(0.035)	(0.001)
Numéraire	-0.003	-0.007	0.004	-0.010	-0.001	0.000	-0.000	-0.005	0.003	-0.002	**-1.054**
	(0.115)	(0.126)	(0.164)	(0.108)	(0.092)	(0.121)	(0.126)	(0.103)	(0.144)	(0.112)	(0.011)

Notes: Bootstrapped standard errors (500 replications) are in parentheses.

Table S3. Estimated Compensating Variation (CV) assuming incomplete pass-through of taxes/subsidies in prices, by income quintiles

	Pass Through			
	75% Taxes /Subsidies		75% Taxes/50% Subsidies	
	USD	% of Income	USD	% of Income
Policy 1: 18% Tax on Junk Foods + 5% additional tax on SB				
Quintile 1	3.27	0.75	3.27	0.75
	(0.06)	(0.01)	(0.06)	(0.01)
Quintile 2	4.52	0.48	4.52	0.48
	(0.08)	(0.01)	(0.08)	(0.01)
Quintile 3	5.73	0.39	5.73	0.39
	(0.10)	(0.01)	(0.10)	(0.01)
Quintile 4	7.53	0.33	7.53	0.33
	(0.12)	(0.01)	(0.12)	(0.01)
Quintile 5	11.85	0.20	11.85	0.20
	(0.20)	(0.00)	(0.20)	(0.00)
Policy 2: 19% Fruits/Vegetables VAT Reduction				
Quintile 1	-4.01	-0.92	-2.67	-0.61
	(0.07)	(0.02)	(0.05)	(0.01)
Quintile 2	-5.00	-0.53	-3.34	-0.35
	(0.09)	(0.01)	(0.06)	(0.01)
Quintile 3	-5.67	-0.39	-3.78	-0.26
	(0.10)	(0.01)	(0.07)	(0.00)
Quintile 4	-6.56	-0.29	-4.37	-0.19
	(0.12)	(0.01)	(0.08)	(0.00)
Quintile 5	-9.36	-0.16	-6.24	-0.11
	(0.18)	(0.00)	(0.12)	(0.00)
Policy 3: Tax and Subsidy Together				
Quintile 1	-0.74	-0.17	0.60	0.14
	(0.07)	(0.02)	(0.06)	(0.01)
Quintile 2	-0.48	-0.05	1.19	0.13
	(0.09)	(0.01)	(0.08)	(0.01)
Quintile 3	0.06	0.00	1.95	0.13
	(0.12)	(0.01)	(0.10)	(0.01)
Quintile 4	0.97	0.04	3.16	0.14
	(0.14)	(0.01)	(0.12)	(0.01)
Quintile 5	2.49	0.04	5.61	0.10
	(0.23)	(0.00)	(0.20)	(0.00)

Notes: Bootstrapped standard errors (500 replications) are in parentheses. Positive values denote welfare losses/tax burdens whereas negative values denote welfare gains/subsidy transfers.
